# Assessment of public awareness and perspectives towards adverse drug reaction reporting system in Karachi, Pakistan

**DOI:** 10.1371/journal.pone.0318139

**Published:** 2025-02-10

**Authors:** Wajiha Iffat, Sadia Shakeel, Shagufta Nesar, Ambreen Qamar, Zille Huma, Hina Rehman, Mahwish Akhtar, Syed Ahsan Ali, Mohammad Nadeem Ansari, Muhammad Yaqoob, Areeb Bin Tariq

**Affiliations:** 1 Department of Pharmaceutics, Faculty of Pharmaceutical Sciences, Dow College of Pharmacy, Dow University of Health Sciences, Karachi, Pakistan; 2 Department of Pharmacy Practice, Faculty of Pharmaceutical Sciences, Dow College of Pharmacy, Dow University of Health Sciences, Karachi, Pakistan; 3 Jinnah College of Pharmacy, Sohail University, Karachi, Pakistan; 4 Department of Physiology, Dow University of Health Sciences, Karachi, Pakistan; 5 Department of Internal Medicine, Faculty of Internal Medicine, Dow University of Health Sciences, Karachi, Pakistan; 6 Department of Pharmacy Practice, Institute of Pharmaceutical Sciences, Jinnah Sindh Medical University, Karachi, Pakistan; 7 Department of Pharmaceutical Chemistry, Faculty of Pharmaceutical Sciences, Dow College of Pharmacy, Dow University of Health Sciences, Karachi, Pakistan; 8 Business Development- Clinical Services Company–ClinRè, United States of America; 9 Dow Institute of Nursing and Midwifery, Dow University of Health Sciences, Karachi, Pakistan; Riphah International University, PAKISTAN

## Abstract

**Background:**

Public involvement in reporting adverse drug reactions (ADRs) generates a broader database on drug safety. Underreporting remains a hindrance to implementing an effective pharmacovigilance system that ultimately affects public health. Hence, it is critical to appraise the public’s awareness of ADR reporting and pharmacovigilance to address the gaps for the enhancement of ADR reporting rate.

**Objectives:**

The current study explored public knowledge and attitudes toward ADR reporting in Karachi, Pakistan.

**Methods:**

A quantitative cross-sectional study was conducted from 3^rd^ Jan 2022 to 30^th^ Nov 2022 using a forty-item questionnaire to evaluate public insights regarding the ADR and its reporting. Descriptive analysis was executed to determine frequencies and percentages for the respondents’ baseline characteristics and the responses toward ADR reporting. The chi-square test (χ^2^) was applied to determine the association between the dependent and independent variables considering a p-value < 0.05 as statistically significant.

**Results:**

The response rate of the present study was 78.3%. More than 80% of the respondents deemed that ADR occurs only with high doses of medicines and over-the-counter medications do not cause any ADR. More than 75% of the respondents did not know that the ADR reporting form is available on the Drug Regulatory Authority of Pakistan (DRAP) website; the response varied significantly with the education *(p = 0*.*002)* and social status *(p = 0*.*0001)* of the respondents. More than 50% of the participants refused to ever report an ADR to health professionals. Physicians (n = 364; 47.7%) and pharmacists (n = 253; 33.1%) were the respondents’ professed most reliable sources to whom ADR can be reported; responses varied significantly with their education *(p = 0*.*003)* and age *(p = 0*.*001)*.

**Conclusions:**

The study has provided insight into the challenges and gaps needed to improve ADR reporting in Pakistan. The outcomes revealed that the public is aware of the benefits of reporting ADRs; however, they do not realize their role and the potentially significant impact on the healthcare system by contributing to ADR reporting. Therefore, it is a need of time to educate the public on the value of reporting ADRs and implement user-friendly and accessible ADR reporting systems in patient care areas to facilitate easier reporting.

## Introduction

World Health Organization (WHO) defines an Adverse Drug Reaction (ADR) as “a response to a drug which is noxious and unintended, and that occurs at doses normally used for the prophylaxis, diagnosis, or therapy of disease, or the modification of physiological function” [1]. It is one of the major public health issues affecting all age groups resulting in an increased rate of morbidity and mortality and adding more economic burden to the already compromised health system [2]. The frequency of ADR in all hospitalizations fluctuates from 4.6 to 17.6% and approximately 80% of the unnecessary medical expenses are due to ADRs [3]. One of the great challenges that causes significant health crises leading to extensive fiscal burden on society is ADR underreporting. The pharmacovigilance system facilitates the detection, assessment, and prevention of long-term and short-term adverse effects of medicine. This system can be strengthened when ADR data is shared timely on signal detection for the identification of previously unknown medicine-related safety issues [4].

The European Directive on Pharmacovigilance has endorsed the integration of patient reporting as it offers several advantages [5]. The reports from consumers offer unbiased perspectives, devoid of the prescriber’s influence, thus furnishing valuable insights into the causality assessment of ADRs. Moreover, it unequivocally delineates the repercussions on individuals’ lives, familial dynamics, and professional engagements. Many countries like the US, Canada, Australia, and New Zealand have established direct patient ADR reporting within pharmacovigilance frameworks, although numerous nations still lack adequate mechanisms for such reporting [6].

Pakistan’s pharmacovigilance system is still in its infancy, though the government has proposed various reforms to enhance the system. The National Drug Policy of Pakistan mentioned the establishment of a drug monitoring and surveillance system in 2003 [7]. In 2012, a pharmacovigilance section was developed by the Drug Regulatory Authority of Pakistan (DRAP) Act, 2012, in the Division of Pharmacy Services upon the orders of the Supreme Court of Pakistan, and the first pharmacovigilance center was established in Punjab [8]. DRAP has framed strategies for pharmacovigilance activities and its provincial drug control unit is issuing drug safety alerts regularly [7]. This was a result of a tragedy that occurred at the Punjab Institute of Cardiology where more than two hundred individuals died after the administration of contaminated medicine (Isosorbide mononitrate 20 mg tablet, batch number J093). Reports acknowledged by the provincial pharmacovigilance center in Lahore were sent to the WHO’s Uppsala Monitoring Centre in Sweden [8].

One of the basic methods for collecting information that forms the core of the global WHO database is the ADR Spontaneous reporting system [9]. This system facilitates the passive data collection of adverse post-marketing risks and events that remained unforeseen during preliminary evaluation. However, the presence of a system is not enough to ascertain system functionality. The contribution of every stakeholder is of major significance in ADR reporting. The inclusion of healthcare professionals is mandatory and augments the medication safety for the population. Despite the benefits, underreporting remains a hindrance to the implementation of an effective pharmacovigilance system and ultimately harms public health. Studies have recognized the critical role of public participation in pharmacovigilance and ADR direct reporting [10, 11]. ADRs reported by the public endorsed those ADRs that are reported by healthcare professionals and contribute to generating new and novel safety signals [12]. Hence, the involvement of patients or consumers in reporting ADRs has become increasingly recognized and implemented in more than 40 countries worldwide [13].

In Pakistan, ADR reporting has been done by physicians and pharmacists [14, 15], and no participation has been observed by the general public as reported in other studies [10, 16, 17]. The patient involvement in reporting generates a broader database of knowledge on drug safety and contributes to earlier ADR detection in contrast to physicians and other healthcare experts. To enhance the reporting rate, it is critical to know the reason behind the suboptimal public participation in ADR reporting that can assist in improving their contribution to ADR reporting and pharmacovigilance. Therefore, the present study was intended to evaluate the knowledge and awareness of the public towards ADR reporting in Karachi, Pakistan.

## Methodology

### Study design and setting

A quantitative, cross-sectional study was conducted in Karachi from 3^rd^ Jan 2022 to 30^th^ Nov 2022 targeting the general public, to evaluate their insights regarding the ADRs and their reporting practices. Respondents were included in the study if they met the following criteria: they were over 18 years old, understood either English or Urdu, were not employed in any healthcare profession, had taken medication at some point in their lives, and were willing to voluntarily participate. However, the respondents who did not provide consent to participate were excluded. The respondents were assured about the confidentiality of their personal information and responses. The current study follows the Strengthening the Reporting of Observational Studies in Epidemiology (STROBE) reporting guideline [18].

### Study instrument

A questionnaire was developed that contains closed-ended questions after going through similar published studies [15, 19, 20]. The survey questionnaire comprised forty questions including 7 questions related to respondents’ demographic characteristics. Ten questions were to probe the respondents’ knowledge of ADRs and their reporting having an option of ‘yes’, ‘no’ and ‘maybe’, each correct option given one point. Incorrect answers and unanswered questions were given zero points. The overall knowledge score was determined by adding together all 10 questions. Based on the overall scores the respondents’ knowledge levels were classified as weak (Score: 0–4), moderate (Score: 5–7), or good (Score: 8–10).

Ten questions were inquisitive of respondents’ attitude toward the importance of ADR reporting using a 5-point Likert scale ranging from 1  =  ‘strongly disagree’, to 5  =  ‘strongly agree’ to show the respondents’ level of agreement with the questionnaire item. Furthermore, the response (1  =  ‘strongly disagree’, 2  =  ‘disagree’) was considered as a negative attitude revealing the respondents’ reluctance to report ADR and (4  =  ‘agree’ and 5  =  ‘strongly agree’) was labeled as a positive attitude showing their optimistic and trustworthy behaviors. Six questions were to probe the respondents’ self-perceived experiences and practices towards ADRs reporting having an option of ‘yes’, ‘no’ and ‘maybe’. Besides, the last section included questions that had multiple choice options regarding respondents’ perceived examples of ADRs, their sources of ADR information, reasons for not reporting ADR, and their perceived most effective way to educate the patient. The respondents were given the choice to select all options that apply.

The developed questionnaire was assessed for its face and content validity by five subject specialists from Dow University of Health Sciences to ensure clarity, relevance, sequences of questions, and compliance; after which the compulsory modifications were made. The questionnaire was also pilot-tested on 30 individuals who were later excluded from the study. Cronbach’s alpha of the modified questionnaire was found to be 0.78.

The English version of the questionnaire was translated into the Urdu language by a person who was proficient in both English and Urdu languages and was blinded to the study. Another healthcare professional who was blinded to the study conducted the back translation from Urdu to English. Particular focus was given to important terms associated with technical and language equivalency. Personalized email invitations and social media posts were used to reach the target audience for an online survey by the researcher and co researchers. By providing respondents with the assurance that their answers would remain anonymous, trust was fostered and their contributions were encouraged. A brief note that highlighted the goal of the study and how their contributions could lead to significant advancements in ADR reporting and pharmacovigilance was sent out to promote their involvement in the study and reduce the possibility of response bias. The invitation included a link to the survey, which respondents could access and fill out online whenever it was convenient for them. The survey was disseminated across diverse platforms to reach a wider audience and make sure it works on a range of devices to combat selection bias. A simple, user-friendly design was used to ensure that the data collection process was easy and smooth.

### Study sampling and sample size

The sample size was estimated using the Raosoft sample size calculator utilizing a confidence level of 95% with a 5% margin error; the sample size was found to be 384 [21]. However, during the data collection, additional data was collected due to feasibility and prompt response rate; therefore, an adjustment was made to the required sample size. Hence, a total of 974 questionnaires were distributed during the period of study. The approach of convenience and snowball sampling was used for the study.

### Data analysis

Descriptive analysis was executed to determine frequencies and percentages for the respondents’ baseline characteristics and the responses towards ADR reporting using Statistical Package for the Social Sciences (SPSS) version 25. Chi-squared test (χ^2^) was conducted to determine whether an association exists between the dependent and independent variables (a p-value < 0.05 was considered statistically significant).

### Ethical consent

The exemption for ethical approval was obtained from an independent institutional review board of Dow University of Health Sciences with protocol # IRB 2101/DUHS/EXEMPTION/2021/650. A section on consent was added to the questionnaire in which the respondents were briefly informed about the goals of the study and were inquired about their consensus to voluntarily participate in the study. In this way, the written consent was obtained from them before the start of the study.

## Results

### Respondents’ demographic information

Among the distributed survey link, 763 forms were filled appropriately with a response rate of 78.3% (*n* = 763). The mean age of the respondents was 36.5 ± 7.5 years. A large proportion of the respondents (n = 562; 73.6%) were females (Table 1). The majority of the respondents (n = 544; 71.2%) were single. More than 90% of the respondents lived in urban settings and around 60% were public or private sector employees. The respondents (n = 354; 46.3%) belonged to the lower middle class.

**Table 1:** Characteristics of the study population.

Characteristics of study population	Frequency (%)
**Age (years)**	36.5 ± 7.5
**Gender**	
Male	201(26.3)
Female	562(73.6)
**Marital status**	
Single	544(71.2)
Married	219(28.7)
**Living setting**	
Urban area	708(92.7)
Rural area	55(7.2)
**Education**	
Inter	155(20.3)
Graduation	441(57.7)
Masters	121(15.8)
Others	46(6)
**Occupation**	
Public or private sector employee	451(59.1)
Entrepreneur	103(13.4)
Students	188(24.6)
Others	21(2.7)
**Social status**	
Lower class	96(12.5)
Lower middle class	354(46.3)
Upper middle class	278(36.4)
Upper class	35(4.5)

### Respondents’ knowledge of ADRs and their reporting

**Fig 1:**
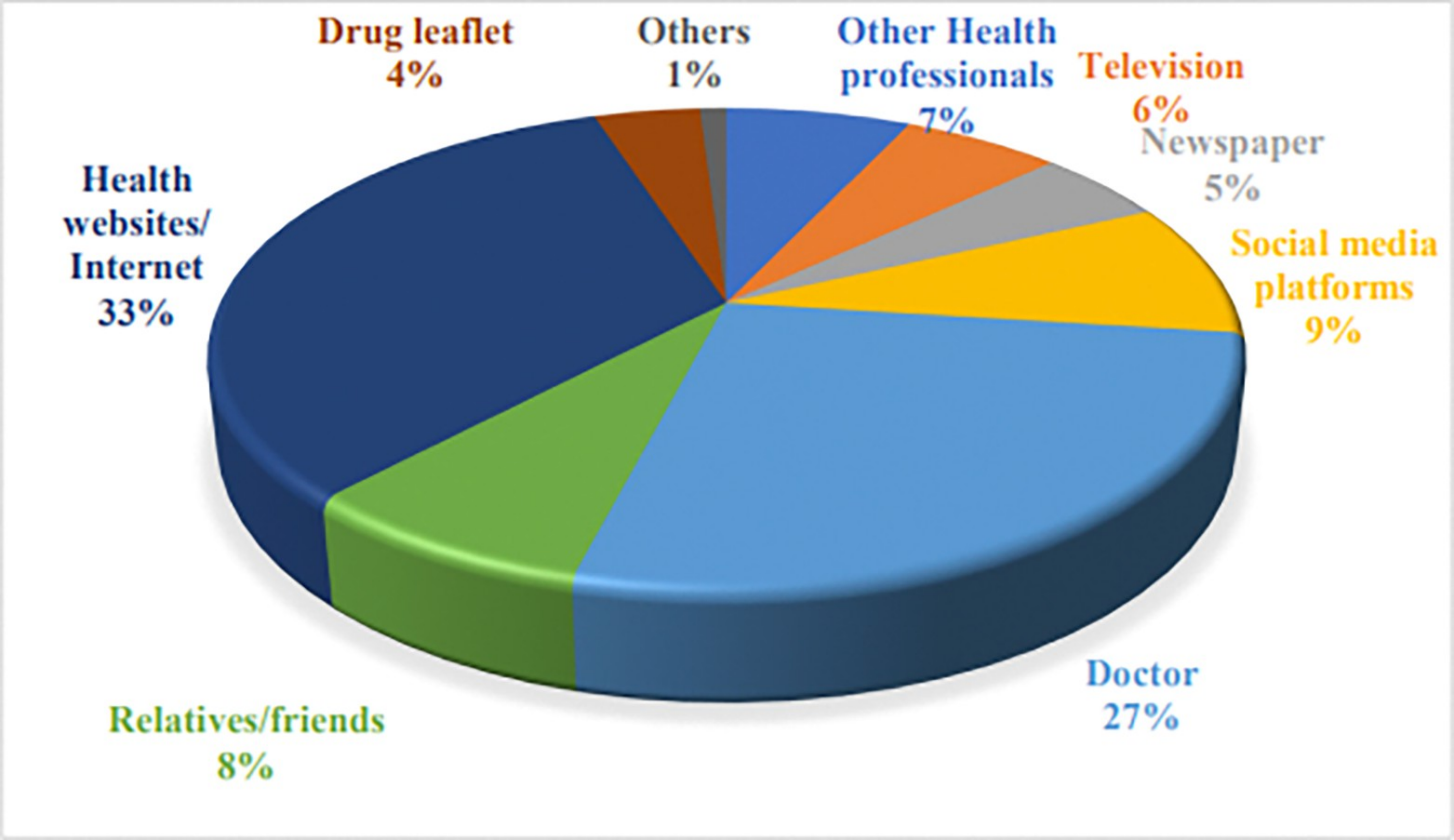
Respondents’ preferred sources for ADR information.

More than 90% of the respondents stated that they knew about the ADRs (Table 2). On inquiring about the description of an ADR; the major response was an unexpected reaction from the medication being used (n = 403; 52.8%), any unknown effect of medication being used (n = 218; 28.5%) whereas n = 142; 18.6% of respondents could not describe ADR. However, the response was significantly associated with the education status *(p = 0*.*003)* and occupation *(p = 0*.*005)* of respondents. More than 80% of the respondents deemed that ADR occurs only with high doses of medicine and over-the-counter medications do not cause any ADR; however, the response was significantly associated with their education *(p<0*.*001)*. The respondents n = 310(40.6%) knew how to report ADRs; responses varied significantly with the gender *(p = 0*.*004)*, age *(p = 0*.*002)* and education *(p = 0*.*0001)* of respondents. More than 75% of the respondents did not know that the ADR reporting form is available on the DRAP website; the response varied significantly with the education *(p = 0*.*002)* and social status *(p = 0*.*0001)* of the respondents. On inquiring about the examples of ADRs; the major responses were allergy-like symptoms/ itching/ rashes (22%), GI upset (18%), developing diarrhea after taking an antibiotic (14%), sleepiness/drowsiness (8%), and nausea/vomiting (7%). Health websites/internet (33%), physicians (27%), and other social media platforms (9%) were the respondents’ observed most reliable sources of information about ADRs of medications (Fig 1).

**Table 2:** Respondents’ knowledge of ADRs and their reporting.

Statement	Correct Response (%)
Do you know what adverse drug reactions (ADR) are?	701(91.8)
Do you think all drugs are safe?	615(80.6)
Do you know that all drugs have ADRs?	587(76.9)
Do you know that ADR may occur at a normal drug dose?	139(18.2)
Do you know that over-the-counter medicines may cause ADR?	150(19.6)
Do you know that herbal drugs may cause ADR?	89(11.6)
Do you know of any drug that has been banned due to ADRs?	361(47.3)
Do you know where to report ADR?	397(52)
Do you know how to report ADR?	310(40.6)
Do you know that the ADR reporting form is available on the Drug Regulatory Authority of Pakistan website?	173(22.6)

### Respondents’ attitude toward the importance of ADR reporting

Table 3 depicts the respondents’ attitudes towards the importance of ADR reporting. More than 80% of respondents comprehend the significance of the ADR reporting and considered it beneficial for the public. Around 85% of respondents opined that educating patients on ADR reporting is essential. Around 80% of those surveyed propounded that healthcare professionals (physicians, pharmacists) should deliver information about the ADRs of prescribed medications. More than 75% of respondents stated that the relevant healthcare authorities should host public events or lectures and that healthcare professionals should be periodically trained to raise public awareness about the need to report ADRs; response varying significantly with the education *(p = 0*.*002)*, and social status *(p = 0*.*006)* of respondents.

**Table 3:** Respondents’ attitude towards the importance of ADR reporting.

Statement	Strongly agree/agree	Neutral	Disagree/Strongly disagree
Do you think that the ADR reporting system can improve patient safety?	591(77.4)	99(12.9)	73(9.5)
Do you think that the ADR reporting system benefits the public?	627(82.1)	118(15.4)	18(2.3)
Do you think that the ADR reporting system should be improved in Pakistan?	619(81.1)	92(12)	52(6.8)
Do you think that educating patients on ADR reporting is essential?	643(84.2)	45(5.8)	75(9.8)
Do you think that health professionals should provide information about the ADR of medication that is prescribed?	625(81.9)	81(10.6)	57(7.4)
Do you think that health professionals should be provided with regular training to educate the public on ADR reporting?	598(78.3)	75(9.8)	90(11.7)
Do you think that the corresponding departments should organize public events or lectures highlighting the significance of reporting ADRs?	589(77.1)	102(13.3)	72(9.4)
Do you think that the government should compel healthcare providers to record ADRs in patients?	544(71.2)	117(15.3)	102(13.3)
Do you think that the government should provide avenues for patients to directly report ADRs?	561(73.5)	127(16.6)	75(9.8)
Do you think that the government should make vigilance on reasons for underreporting?	412(53.9)	210(27.5)	141(18.4)

### Respondents’ self-perceived experience and practice towards ADRs and their reporting

Half of the respondents negated ever experiencing an ADR and never report any ADR to a health care professional (Table 4). The majority of the respondents (n = 623; 81.6%) were keen to know about possible ADRs that may occur due to the medicine they consume whereas (n = 510; 66.8%) respondents stated to report an ADR if they will ever experience it. The respondents (n = 512; 67.1%) had asked the health care professional about possible ADR they may experience from a medication. On inquiring about the respondents’ actions whenever they experienced an ADR; the major responses observed were to do nothing because it was tolerable (29%) and to stop the drug (23%). Physicians (n = 364; 47.7%) and pharmacists (n = 253; 33.1%) were the respondents’ professed most reliable sources to whom ADR can be reported; responses varied significantly with their education *(p = 0*.*003)* and age *(p = 0*.*001)*.

**Table 4:** Respondents’ self-perceived experience and practice towards ADRs and their reporting.

Statement	Yes (%)	No (%)	Maybe (%)
Have you ever experienced an ADR to any medication you took?	291(38.1)	391(51.2)	81(10.6)
Have you ever reported an ADR you experienced with any drug to a healthcare professional?	279(36.5)	397(52)	87(11.4)
If you ever experience an ADR, would you report it?	510(66.8)	145(19)	108(14.1)
Are you keen to know about possible ADR that may be due to the medicine you consume?	623(81.6)	95(12.4)	45(5.8)
Have you ever asked the health care professional about possible ADR you may experience from a medication?	512(67.1)	215(28.1)	36(4.7)
Did your doctors or other health professionals ever provide you with information about the ADR of the medication you are taking?	505(66.1)	218(28.5)	40(5.2)

**Fig 2:**
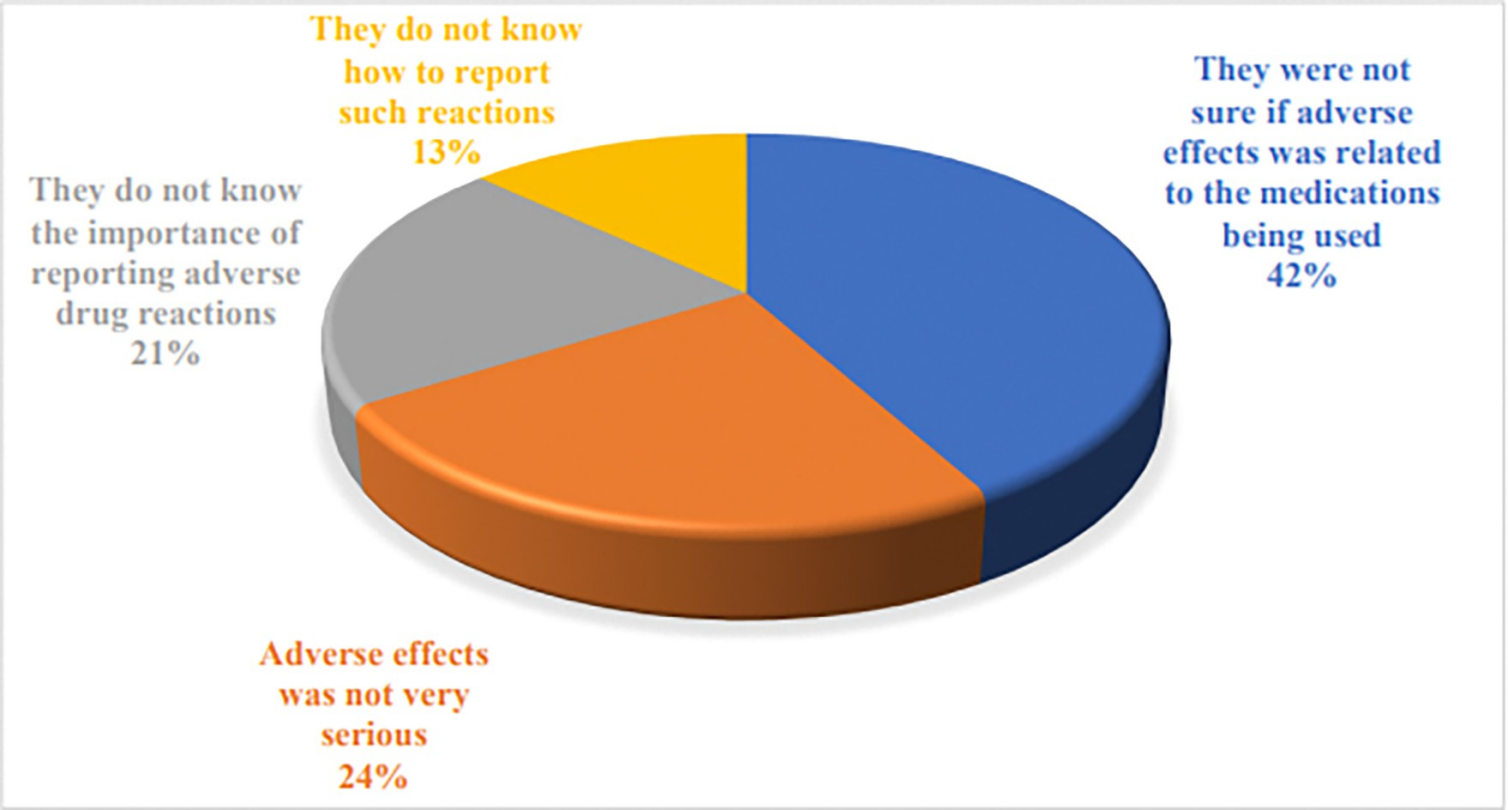
Respondents’ perceived barriers to reporting their own ADR experiences.

**Fig 3:**
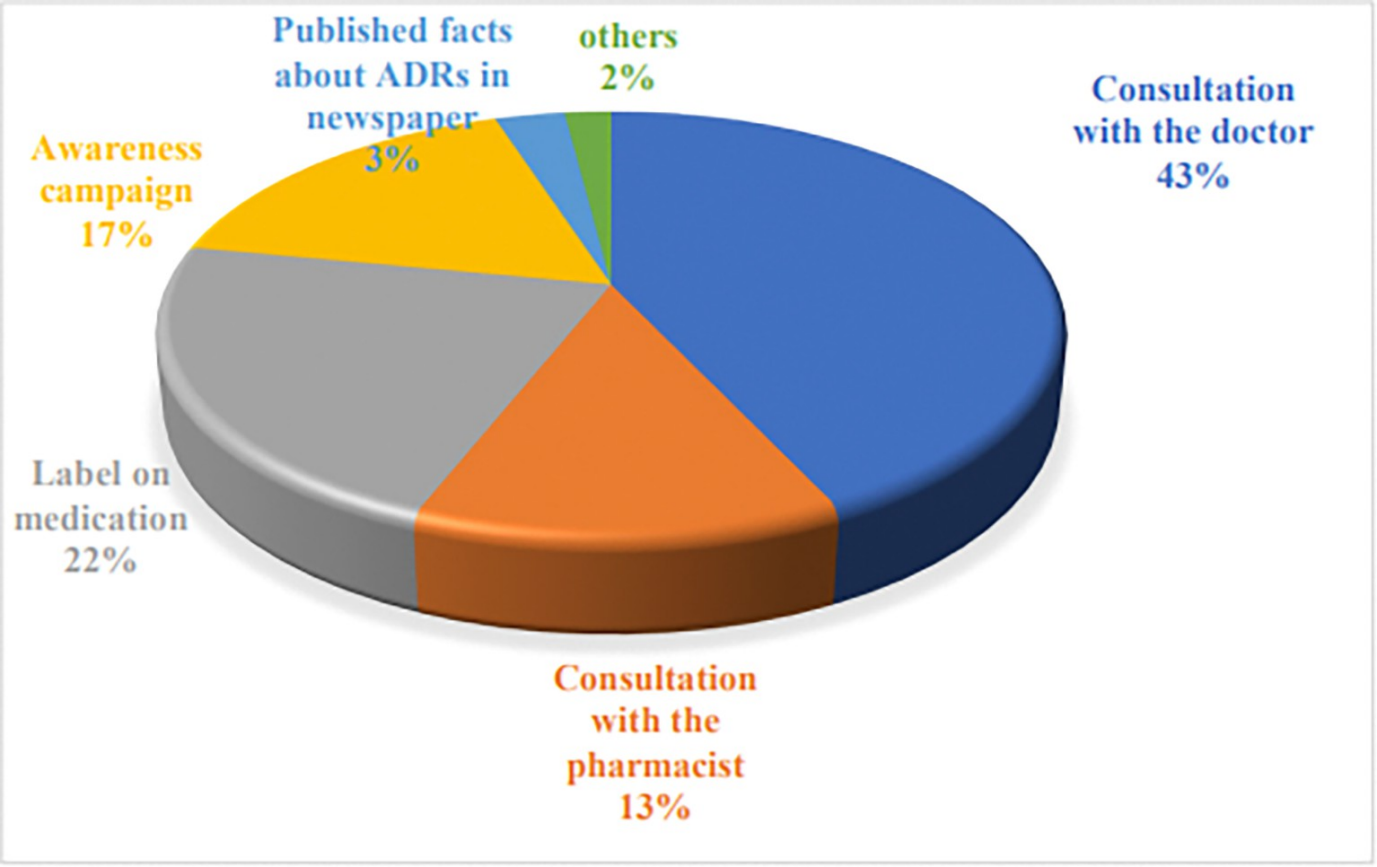
Respondents’ preferred approach to enhance ADR reporting.

Fig 2 depicts the respondents’ perceived barriers to reporting their own ADR experiences. The major response was they were not sure if adverse effects were related to the medications being used (42%). Fig 3 depicts the respondents’ preferred approach to enhance ADR reporting. The major responses were the consultation with the physicians (43%) to educate the patient, the label on medication in comprehensible language (22%), and an awareness campaign (17%). Thirty-two percent of the respondents opined that the most applicable method for reporting ADR is to directly communicate ADR to healthcare professionals. The other responses include through phone by calling or text message (30%) and an online system of reporting ADR (27%).

## Discussion

The findings of the present study revealed that the public knew about ADR reporting; however, they do not recognize their role and the potentially significant impact on the healthcare system by contributing to ADR reporting. The literature review highlighted the fact that no such study has been carried out to determine the general public knowledge about ADR and its reporting system in Pakistan although, many studies have been carried out to determine the knowledge and opinion of healthcare professionals [15, 22]. There is no denying the fact that patients are well familiar with their health and the adverse effects can be reported by patients as soon as they become apparent, which aids in the early identification of possible medication safety issues. Hence, they are in a better position to contribute to ADR reporting that can ultimately improve drug safety [11]. The current study showed a response rate of 78.3% which is relatively better than the studies conducted in different countries [23, 24] although fewer studies showed a better response rate of 90.7% [25, 26]. A lack of interest in the subject or general reservations about online surveys may cause certain individuals to disregard or reject the survey request.

The outcomes of the present study highlighted the fact that the public understood the benefit of ADR reporting and showed interest in gaining further knowledge but did not know how they could actively participate in the ADR reporting procedure. There exists a significant gap in understanding how to report them which is likely to be affected by sociocultural and educational factors. Limited health literacy is one of the primary factors that hinders the comprehension of pharmacovigilance concepts in populations with lower socioeconomic status. Additionally, there are misconceptions such as the belief that ADRs are limited to high-dose medications, further exacerbate underreporting. In Pakistan, cultural norms are such that patients do not question healthcare professionals which discourages patients from discussing ADRs openly. Public health interventions should prioritize culturally sensitive strategies, such as simplified reporting mechanisms and awareness campaigns targeted at communities with low literacy levels. Our findings were comparatively better than those observed in another study where only 25.7% of the respondents understood the description of ADR [23]. Another study’s results were found to be slightly better where 68.01% of participants knew about ADR meaning [24]. In the present study, results showed eighty percent of the respondents negated that all drugs are safe while contradictory findings were reported in another study where 89.98% of respondents considered that all marketed drugs are safe [24]. It was alarming to note that the majority of the respondents contradicted that herbal drugs may cause ADR. The consumption of herbal drugs and herbal products has increased in different disease conditions with the general perception that “natural drugs are safe” and alleged satisfaction with therapeutic outcomes in contrast to conventional products [22]. This public perception is untrue and misleading and has been shown to produce various undesirable reactions, several toxic effects, drug interactions, and even incidences of morbidity or mortality [27].

A considerable portion of respondents in the current study depicted poor participation in reporting that may result from inadequate or ambiguous information regarding where and how to submit ADRs. People may be discouraged from reporting ADRs if they believe the process is difficult or time-consuming and they might think that their particular reports won’t have any impact [30]. It was also observed in the present study that the majority of the respondents did not know that the ADR reporting form was available on the DRAP website. ADRs can be reported by the public and medical practitioners using the DRAP web platform. Although digital platforms offer great potential for improving ADR reporting, technological barriers such as limited internet access and digital literacy remain significant challenges in Pakistan. Offline options, like paper-based reporting forms distributed at pharmacies and hospitals, should complement digital systems to ensure accessibility for all populations. Reports can be sent directly to the regulating body; based on these reports the regulatory authority could take appropriate measures to assure drug safety [10]. Similar findings were observed in other studies that the respondents were unaware of the way by which the ADR could be reported [13, 23, 28]. It is widely reported that the general population indicated uncertainty about ADR reporting, the existence of reporting centers in their vicinity and the availability of forms on the website [24]. Patients’ motivation to report ADR might rise when they get reassurance that reports are valued and helping to improve drug safety [32]. Empowering patients through education and incentives can significantly improve ADR reporting rates. Pharmacovigiliance systems can be improved by integrating patient-centric digital tools, such as mobile applications with user-friendly interfaces which could simplify the reporting process. The incorporation of public recognition or small incentives for ADR reports may encourage greater participation and engagement in pharmacovigilance systems [33].

In the present study, the majority of the respondents negated reporting an ADR experience to a healthcare professional. Few suppositions why patients don’t report depend upon the patients’ consideration regarding whether the adverse reaction was due to the medication intake or not, or due to the unawareness about the existence of a pharmacovigilance center or patient perception about the significance of ADR reporting. The current findings highlight the pressing need for specific measures to address the challenges in ADR reporting. These could be overcome by integrating ADR reporting and pharmacovigilance in the curricula for medical and pharmacy students foster ADR education among the masses. DRAP and healthcare institutions should establish ADR reporting booths such as dedicated desks in hospitals and pharmacies to facilitate in-person ADR reporting. ADR reporting can be encouraged by utilizing social media campaigns for mass education and the process of reporting should be simplified using mobile apps and online portals. The suggested measures are effective globally for the enhancement of ADR reporting systems. Besides, patients might be concerned that disclosing an ADR may have unfavorable effects on their relationships with medical professionals [11].

The majority of respondents in the present study showed keen interest in seeking information about the possible ADRs from healthcare professionals; however, only 66.1% of the respondents stated that they had ever received any information from their physicians or other health professionals about the ADRs of the medication. A study conducted in Korea showed comparatively better results [29]. Meanwhile, in another study, only 39% of respondents received information about the side effects of medication from pharmacists [24]. In our study, physicians and pharmacists were the respondents’ most reliable professionals to whom ADR can be reported. Healthcare providers could serve as patients’ liaisons, assisting them in reporting ADRs to the regulatory authority. Many studies have endorsed that pharmacists are instrumental in imparting ADR education and reporting on account of their accessibility and trustworthiness [7, 14, 15]. They can proactively educate patients about potential ADRs at the point of dispensing and assist in reporting through official channels. In Pakistan, the ADR reporting frameworks can be strengthened through pharmacist-led interventions that have proven to improve public health outcomes by enhancing public awareness and their willingness to report ADRs. Furthermore, their roles can be expanded through the provision of targeted training programs and public health campaigns for improved ADR reporting. Both patient knowledge and willingness to report ADRs are increased when healthcare providers actively inform and urge patients to do reporting. A similar finding was observed in a study that showed 40% of respondents believed physicians are the most appropriate healthcare professionals to whom ADR can be reported [24]. One study reported that respondents strongly rely on physicians (61.8%) to inquire more about ADR followed by pharmacists (36.8%) and the internet(18.6%) [23]. A study conducted in Riyadh showed that the respondents stated to notify physicians(82.8%), pharmacists(79%) and pharmacovigilance centers (55.8%) about the occurrence of ADR [23]. However, it is observed that ADR detection was improved when pharmacists were approached in contrast to physicians [20]. These findings are further endorsed in a study that emphasized the recognition and significance of the pharmacist’s role in ADR reporting [30, 31].

In the present study, the respondents concurred that the public benefits from the ADR reporting and the system needs to be improved in Pakistan. The parallel optimistic approach was observed in different studies whereby the majority of respondents believed that ADR reporting is beneficial [32, 33]. The majority of the respondents indicated that the relevant healthcare authorities should host public events or lectures and that health professionals should be periodically trained to raise public awareness about the need to report ADRs. This finding was endorsed where respondents had higher education and showed an optimistic attitude in contrast to those having primary school level education [34].

Few studies reported that the most common barriers associated with ADR reporting among the general public were unsatisfactory knowledge about the importance of reporting ADR and lack of awareness about the reporting procedures developed by the Ministry of Health [13, 34, 35]. These barriers can be overcome by following and implementing the pharmacovigilance system in countries like Sweden and the Netherlands which have developed accessible online reporting platforms and public education campaigns for swift reporting of ADRs [32, 33]. Incorporating these strategies will help in integration of ADR reporting into Pakistan e health initiatives for better participation and improved system efficiency. DRAP should conduct awareness programs to educate the public on the value of reporting ADRs. Community engagement can be enhanced by improving healthcare professionals’ practices in educating and motivating the public to report ADR, raising awareness through the campaign and utilizing media outreach comprising of TV, radio, newspapers, and social media to spread awareness about the reporting system. Providing small incentives or recognition to those who report can encourage more participation. Additionally, dedicated reporting desks in hospitals and pharmacies should be established. Hence, the campaign strategies should be continuously improved in consideration of the current findings and public opinion. The current finding emphasizes the dire need for robust pharmacovigilance policies in Pakistan. The pharmacovigilance system can be significantly strengthened by addressing public awareness barriers, augmenting the pharmacist’s role in public, and adopting global best practices subsidising reduced healthcare costs and improved public health outcomes.

### Limitations of the study

The current study was limited to Karachi; therefore, the findings cannot be generalized to the entire Pakistani population. As a result, a nationwide survey is proposed to obtain a larger range of responses from the Pakistani public about ADR reporting. The cross-sectional study’s design has the incapacity to evaluate causal correlations. Although, the results obtained from the study can still be used by healthcare professionals and policymakers. Furthermore, as the current study was conducted in the form of online survey; therefore, selection bias could be possible since respondents who have access to the internet or particular social media habits are more likely to participate, which could reduce representativeness. Besides, the results may be skewed by response bias, as the respondents who were more aware about the importance of ADR reporting issues are more likely to reply.

### Future recommendations

Based on the outcomes of the present study, the national pharmacovigilance program should be vigorously enforced by engaging stakeholders including drug manufacturers, medical associations, and patient advocacy groups in pharmacovigilance initiatives to decrease ADR-related morbidity and death. Regulatory authorities should mandate ADR reporting by healthcare professionals and pharmaceutical companies. ADR reporting can be improved by continuous education and training programs for healthcare professionals on the importance of ADR reporting to improve the quality and quantity of reports. Moreover, implementing user-friendly and accessible ADR reporting systems, such as online portals, mobile apps, and dedicated desks in hospitals and pharmacies, can facilitate easier reporting. With this approach, Pakistan’s healthcare system will be revitalized.

## Conclusion

The study has provided insight into the challenges and gaps needed to improve ADR reporting. The outcomes revealed that the public is aware of the benefits of reporting ADRs; however, they do not realize their role and potentially significant impact by contributing to ADR reporting and pharmacovigilance. The regulatory bodies should emphasize educating healthcare professionals and the public about drug safety to lessen the economic burden by instigating them to report ADR. It is therefore need of time to develop an easily accessible, clear, and user-friendly procedure that can be employed to report ADRs.
